# Application of multi-objective optimization in the study of anti-breast cancer candidate drugs

**DOI:** 10.1038/s41598-022-23851-0

**Published:** 2022-11-11

**Authors:** Yuan Mei, Kaijun Wu

**Affiliations:** School of Electronics and Information Engineering, Lanzhou Jiaotong University, Lanzhou, China

**Keywords:** Data mining, Virtual drug screening

## Abstract

In the development of anti-breast cancer drugs, the quantitative structure-activity relationship model of compounds is usually used to select potential active compounds. However, the existing methods often have problems such as low model prediction performance, lack of overall consideration of the biological activity and related properties of compounds, and difficulty in directly selection candidate drugs. Therefore, this paper constructs a complete set of compound selection framework from three aspects: feature selection, relationship mapping and multi-objective optimization problem solving. In feature selection part, a feature selection method based on unsupervised spectral clustering is proposed. The selected features have more comprehensive information expression ability. In the relationship mapping part, a variety of machine learning algorithms are used for comparative experiments. Finally, the CatBoost algorithm is selected to perform the relationship mapping between each other, and better prediction performance is achieved. In the multi-objective optimization part, based on the analysis of the conflict relationship between the objectives, the AGE-MOEA algorithm is improved and used to solve this problem. Compared with various algorithms, the improved algorithm has better search performance.

## Introduction

Breast cancer is the most common cancer in women worldwide. From the 2020 edition of the Global Cancer Statistical Report released by the International Agency for Research on Cancer in February 2021^1^, we can know that the prevalence of female breast cancer has surpassed that of lung cancer in 2020, becoming the cancer with the highest incidence of cancer in the world. In China, breast cancer ranks fourth in the national cancer incidence, second only to lung cancer, colorectal cancer and gastric cancer^2^. Therefore, the development of anti-breast cancer drugs is of great significance. In the field of medicinal chemistry, many scholars have studied and analyzed a large number of drugs, and found that compounds with antagonistic activity may be candidate drugs for the treatment of breast cancer^3^. When measuring the quality of a candidate drug, the biological activity of the compound ($IC_{50}$, which is usually taken as a negative logarithm and expressed by $PIC_{50}$. The larger the $PIC_{50}$ value indicates the higher the biological activity), the properties of pharmacokinetics and the Safety, collectively known as ADMET (Absorption, Distribution, Metabolism, Excretion, Toxicity) properties, should be considered comprehensively. In clinical experiments, due to the differences between the data obtained on experimental animals and clinical data, it has brought some troubles to researchers. At the same time, considering the high cost of a single experiment, it is not conducive to repeated testing^4^. In recent years, with the development of computers, researchers have gradually used computer models to analyze experimental data^5,6^. By constructing the quantitative structure-activity relationship (QSAR) model to predict new compounds with better biological activity, the research cost is greatly reduced and the research efficiency is improved.

In traditional research methods, linear weighting is often used to explore the relationship between the molecular descriptors of compounds and their biological activity and ADMET properties^7–9^. This is often inefficient, and the model prediction results often have large deviations. In addition, these studies often only consider the relationship between individual molecular descriptors and a single target, ignoring the interaction between a large number of molecular descriptors in actual drug preparation. In recent years, various machine learning algorithms have been widely used in the construction of QSAR models^10–13^, and have achieved good predictive performance. However, many current studies mainly focus on building a relationship model between the molecular descriptors of compounds and their biological activities and their ADMET properties to improve the predictive performance of the models. Lack of exploration of the relationship between molecular descriptor selection process, compound biological activity and AMDET properties, it is impossible to directly select drug candidates.

Therefore, this paper discusses the selection of molecular descriptors, the relationship mapping model between molecular descriptors and their biological activities and five ADMET properties, and multi-objective optimization. A complete selection framework for anti-breast cancer drug candidates was constructed. Specifically, the contributions of this paper are as follows.

- From multiple perspectives, a new feature selection method based on unsupervised spectral clustering is designed. The selected features have less redundancy and more comprehensive information expression ability.
- Based on the analysis of the conflict relationship of six optimization objectives, the AGE-MOEA algorithm^14^ is improved and used to solve the problem. Compared with many algorithms, the improved algorithm achieves better search performance.
- A complete anti-breast cancer candidate compound selection framework was proposed, which provided guidance for the selection of candidate compounds.

## Related work

### Feature selection

Feature selection is the process of selecting independent feature subsets with stronger expression information from the original feature set. Generally, drug candidate data has the characteristics of high-dimensional small samples, and there is often a lot of redundant information. Feature selection not only helps to remove redundant features and reduce the training time of the model, but also helps to improve the prediction performance of the model. Traditional feature selection methods are often based on supervised, such as Relief algorithm^15^, CFS algorithm^16^. However, sample class labeling is often difficult to obtain, so in recent years, unsupervised feature selection algorithms have attracted wide attention from scholars.

Dash et al.^17^ proposed an unsupervised feature selection algorithm based on entropy ranking, which uses information entropy to measure the importance of features, so as to select the optimal feature subset. Hou et al.^18^ proposed a multi-view feature selection method based on adaptive similarity and view weight. By learning the common similarity matrix of different views, the common structure of each view is described. The sparse $L_{2,1}$-norm constraint is used to learn the sparse feature selection matrix. Li et al.^19^ proposed an unsupervised multi-view feature selection method based on similarity matrix learning and matrix correction. Feature selection is embedded in the learning of data manifold structure graph^20^. The two promote each other, which can effectively reduce information redundancy and retain feature correlation. Xie et al.^21^ proposed the idea of unsupervised feature selection based on spectral clustering(FSSC). On the basis of using the adaptive spectral clustering algorithm to cluster the correlation coefficient matrix between features, a feature importance measure method combining feature discrimination and feature independence is proposed to filter the features in the cluster. The experimental results show that FSSC achieves better performance in three unsupervised feature selection methods. However, it is worth noting that FSSC only conducts experiments on small sample data sets with a sample size of about 100, and its performance on this issue is slightly bleak.

Therefore, this paper continues the follow-up work of FSSC, and uses the correlation coefficient, cosine similarity and grey correlation degree between features to mine the hidden layer relationship between features from multiple perspectives. At the same time, after using the spectral clustering algorithm for feature clustering, the sum of the weights of the edges connected to the features in the cluster is used as the measure of the importance of the current features, and the important features are selected.

### Relation mapping

QSAR relationship mapping model is one of the computer-aided tools for drug discovery and design. Through the description information of compounds, the relationship between activity, toxicity and carcinogenicity of compounds is established. In recent years, with the development of machine learning algorithms, the predictive performance of QSAR relationship mapping models has also been improved.

Miler et al.^22^ established and compared 24 predictive models and found that the predictive performance of the nonlinear model was better. Gu et al.^23^ used graph attention network to classify and predict the properties of drug ADMET, and achieved good classification results, but did not discuss the selection of molecular descriptors for optimal compounds, and the overall prediction of their algorithm Performance is low. Xie et al.^24^ used a neural network model to predict and analyze the properties of drug ADMET, and the prediction accuracy was improved to a certain extent, but did not discuss the selection of compounds. Jia^25^ used a variety of machine learning algorithms to build a quantitative prediction model between compounds and molecular descriptors, but lacked an overall consideration of the pros and cons of compound treatments. Xu et al.^26^ combined particle swarm algorithm and machine learning algorithm to predict the biological activity and ADMET properties of the compound respectively, and analyzed the drug mechanism of the compound comprehensively. However, the overall prediction performance of the model is still low, and in addition, the overall consideration of the biological activity and ADMET properties of the compound is lacking, and the task of compound selection cannot be directly performed.

Therefore, this paper combines a variety of machine learning algorithms to experiment, and finally selects CatBoost algorithm^27^ to map the relationship between each other, which achieves higher prediction performance and lays a foundation for the optimization of subsequent compound selection problems.

### Multi-objective optimization

In order to select drugs, on the basis of completing the selection of compound molecular descriptors and the construction of the relationship mapping model, it is necessary to combine the six relationship mapping models to solve the optimization problem, so as to obtain the value range of important molecular descriptors corresponding to the six optimization objectives as good as possible.

Generally, the multi-objective optimization problem can be defined as follows.

1 $$\begin{aligned} \begin{array}{c} \min \limits _{x \in \chi } \quad f(x) = (f_1(x),...,f_m(x))^{T} \\ s.t. \quad g_{i}(x) \le 0, \quad \forall i \in \{1,...,,p\}\\ \quad h_{i}(x) = 0, \quad \forall j \in \{ 1,...,q\}\\ \end{array} \end{aligned}$$

where $\chi$ is the solution space and x is the potential solution; $f_1(x),..., f_m(x)$ are objectives to be optimized, m is the number of objectives to be optimized ; *g*(*x*) and *h*(*x*) are inequality constraint and equality constraint respectively; i and j are the numbers of inequality constraints and equality constraints, respectively.

In solving multi-objective optimization problems, it is an important condition to discuss the conflict between objectives^28^. When there is no conflict between the objectives, the optimal solution can be obtained by optimizing each objective function independently. However, in many problems, there are often both conflict and non-conflict relationships between goals. Therefore, it is necessary to analyze the relationship between the objectives before selecting the multi-objective optimization method. However, in the study of many drug optimization problems, this is often absent. In addition, the Pareto front of the optimization problem is often unknown. For this reason, Abel et al.^28^ proposed to combine the results of various optimization algorithms as the approximation of the Pareto front of the current problem after deduplication and used it to solve the subsequent problems, and achieved ideal evaluation results.

On the basis of determining the conflict relationship between objectives, it is necessary to determine the corresponding optimization algorithm to solve the problem. Because of the high efficiency of genetic algorithm, it has been widely used in solving optimization problems. The NSGA^29^ algorithm retains the excellent individuals in the population through non-dominated sorting, and achieves good solution performance. However, there are problems such as high computational complexity and easy loss of excellent individuals. On the basis of NSGA algorithm, NSGA-2 algorithm^30^ introduces fast non-dominated sorting, crowding distance, elite strategy and other techniques to solve the above problems. However, when the dimension of the target to be optimized is high (usually, we think that the dimension of the target to be optimized is greater than 3, which is a high-dimensional problem), the populations will become non-dominated. Therefore, the effect of NSGA-2 algorithm in solving high-dimensional multi-objective optimization problems will become very unsatisfactory. To this end, NSGA-3^31^ changed the crowding degree distance to the reference point method in the selection process, and achieved better search performance in high-dimensional target optimization problems. Different from the NSGA-2 algorithm approach, MOEA/D^32^ explicitly decomposes the multi-objective optimization problem into several scalar optimization sub-problems defined by a set of weight vectors. Using a steady-state evolution model, an appropriate representation of the Pareto front can be achieved by defining a set of “prior” generated uniformly distributed weight vectors. It has faster convergence speed and lower computational complexity, and the obtained solution distribution is also more uniform. AGE-MOEA^14^ considers the search performance of different Pareto front-end ensemble shapes, and achieves better search performance on multiple tasks. Although the AGE-MOEA algorithm has good local search ability, it has some deficiencies in global search ability, and the diversity of solution results is also poor.

In order to make up for the insufficiency of current research, this paper adds an analysis of the conflict relationship between optimization objectives. At the same time, considering that the Pareto front end of this paper is unknown, Abel et al.^28^ adopted the solution set of various optimization algorithms as the approximation of the Pareto front end of this problem. Finally, this paper adds improvements to the AGE-MOEA algorithm to enhance the search performance of the algorithm. The improved algorithm is used to solve the problem in this paper, and the superiority of the improved algorithm is verified.

## Drug selection framework

### Methods overview

This experiment aims to construct a drug selection framework for optimal molecular descriptors of anti-breast cancer-related compounds. Among them, only when the compound has excellent biological activity and its ADMET properties are at least 3 human-friendly, can it meet the candidate criteria. Therefore, this problem can be regarded as the solution of a constrained nonlinear multi-objective optimization problem. The specific solution process of this paper is as follows:

1. First, a multi-view processing method is proposed to explore the deep relationship between features and the obtained relationship matrix is regarded as a weighted undirected graph. Use spectral clustering algorithm to perform subgraph segmentation and complete feature clustering. The proposed novel feature importance measurement method is used for feature selection to obtain the final candidate feature subset.
2. Then, using the selected feature subset, a variety of machine learning algorithms are used to construct the relationship model between molecular descriptors and compound bioactivity, molecular descriptors and ADMET properties. A CatBoost algorithm based on decision tree and ensemble learning is selected to construct the subsequent optimization objective function.
3. Finally, for the above six optimization objectives, after determining the Pareto front end of this problem, the conflict relationship between the objectives is analyzed. The AGE-MOEA algorithm^14^ is improved, and the improved algorithm is used to solve the final multi-objective optimization problem.

### Data pre-processing

Considering the large amount of redundant data in the original molecular descriptors, the features with more than 90% of the molecular descriptors with 0 value and the features with a correlation greater than 95% were eliminated. In addition, in order to facilitate the subsequent optimization processing, the hERG values in ADMET properties (Caco-2, $CYP_{3}A_{4}$, hERG, HOB, MN) are exchanged between 0 and 1 categories, and category 1 is used to represent the human-friendly properties. . The MN also performs the same process.

### Feature selection

**Figure 1:**
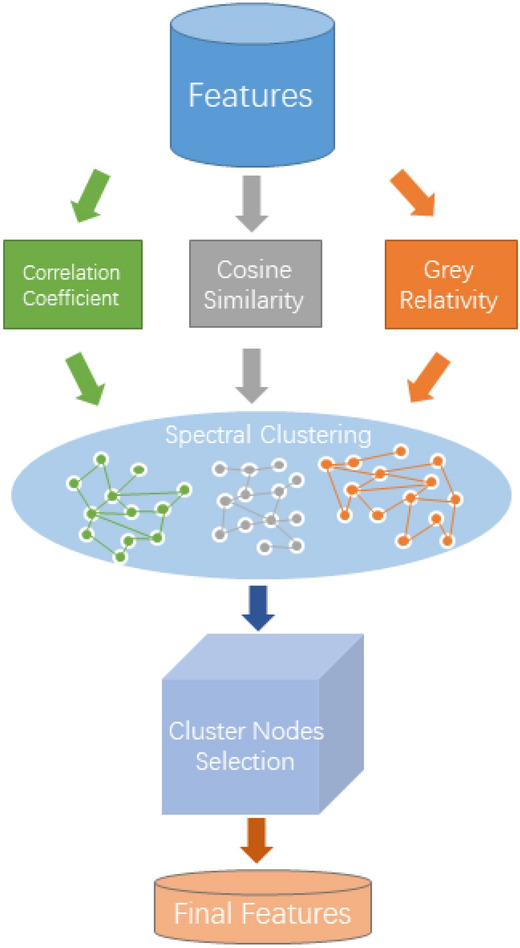
Feature selection flow chart.

In order to improve the versatility of the selected features in solving multiple optimization objectives, from the perspective of similar feature clustering, a multi-perspective-based unsupervised feature clustering selection framework is designed. The specific framework is shown in Fig. 1.

As shown in Fig. 1, in the process of feature selection of the original data, the correlation coefficient, cosine similarity and gray correlation coefficient^33^ are used to explore the relationship between features. The specific calculation formula is as follows.

#### Correlation coefficient

2 $$\begin{aligned} r = \frac{\sum _{i=1}^{n}(x_{i}-{\overline{x}})(y_{i}-{\overline{y}})}{\sqrt{\sum _{i=1}^{n}(x_{i}-{\overline{x}})^{2}}\sqrt{\sum _{i=1}^{n}(y_{i}-{\overline{y}})^{2}}} \end{aligned}$$

where r represents the correlation coefficient between feature x and feature y ; $x_{i}$ represents the ith value of feature x; ${y}$ represents the ith value of characteristic y; ${\overline{x}}$ and ${\overline{y}}$ are the averages of all values of characteristic x and characteristic y, respectively.

#### Cosine similarity

3 $$ cos {{ \varTheta}} = \frac{{\sum\nolimits_{{i = 1}}^{n} {x_{i} *{y} _{i} } }}{{\sqrt {\sum\nolimits_{{i = 1}}^{n} {(x_{i} )^{2} } } \sqrt {\sum\nolimits_{{i = 1}}^{n} {({y} _{i} )^{2} } } }} $$

where, $cos \varTheta$ represents the angle between feature x and feature y; $x_{i}$ represents the ith value of feature x; ${{y} _{i} }$ represents the ith value of characteristic y.

#### Grey correlation coefficient

4 $$\begin{aligned} \rho _{ij}(k) = \frac{\mathop {min}\limits _{i}\mathop {min}\limits _{k}|x_{j}(k)-x_{i}(k)|+\xi \mathop {max}\limits _{i}\mathop {max}\limits _{k}|x_{j}(k)-x_{i}(k)|}{|x_{j}(k)-x_{i}(k)|+\xi \mathop {max}\limits _{i}\mathop {max}\limits _{k}|x_{j}(k)-x_{i}(k)|} \end{aligned}$$

where $\rho _{ij}(k)$ is the grey correlation coefficient of the jth feature and the ith feature on the kth value; $\xi$ is the resolution coefficient, usually 0.5.

#### Feature importance

After the above processing, three matrices measuring the relationship between features can be obtained. They are regarded as a weighted undirected graph, and the spectral clustering algorithm^34^ is used to cluster the features to form feature clusters with different pharmacological properties.

For the final feature selection, a feature selection algorithm based on the representation of the strongest correlation within a cluster is proposed. The specific calculation process is as follows.
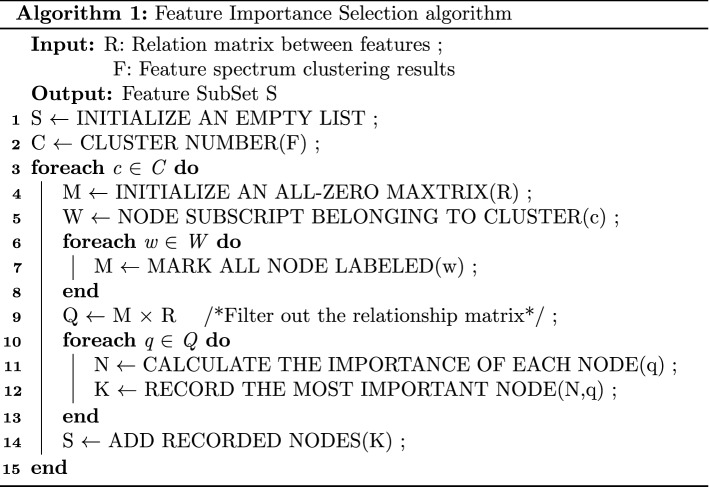


As shown in Algorithm 1, in the process of intra-cluster feature selection, first, each cluster in the clustering results obtained after spectral clustering begins to traverse, and the mask matrix is used to block the feature weights in the feature relationship matrix that do not belong to the current cluster. (That is, feature importance is calculated only within the current cluster.) Then, the node with the largest sum of edge weights in the current cluster is used as the most important feature representation in the current cluster (representing the strongest correlation between the current feature and other features in the cluster.) Then, the most important features in the current cluster are stored in the candidate feature subset. After repeating the above filtering operation for 10 times, features with a frequency greater than 10 are taken as the final features.

### Relation mapping

In the compound selection process, the six objectives of the current compound bioactivity ($PIC_{50}$) and ADMET properties (Caco-2, $CYP_{3}A_{4}$, hERG, HOB, MN) need to be considered simultaneously. Therefore, it is necessary to establish the relationship between molecular descriptors (features) and these six objectives. Among them, the prediction of $PIC_{50}$ value is a regression problem, and the prediction of ADMET properties (Caco-2, $CYP_{3}A_{4}$, hERG, HOB, MN) is a binary classification problem. After comparative analysis with various machine learning algorithms, the CatBoost algorithm^27^ was finally selected to construct the relationship mapping model between them. Among them, CatBoost is a GBDT framework with fewer parameters, support for categorical variables, and high accuracy, implemented with oblivious trees as base learners. He solved the problems of Gradient Bias and Prediction shift, which can effectively reduce the occurrence of over-fitting and improve the accuracy and generalization ability of the algorithm.

Based on the respective feature mapping relationships between the six targets to be optimized ($PIC_{50}$, Caco-2, $CYP_{3}A_{4}$, hERG, HOB, MN) and the selected molecular descriptors. The compound selection process can be expressed as a constrained multi-objective optimization problem shown in Formula 5. (The biological activity and ADMET properties of the compound are as high as possible provided that at least three of the ADMET properties of the compound are beneficial to the human body.)

5 $$\begin{aligned} \begin{array}{c} min \quad H(x) = (-h_1(x),-h_2(x),...,-h_6(x))^{T} \\ s.t. \quad |-h_{2}(x) - ... - h_{6}(x)| \ge 3\\ \end{array} \end{aligned}$$

where $h_{1}(x),...,h_{6}(x)$ represents the six objective functions of $PIC_{50}$, Caco-2, $CYP_{3}A_{4}$, hERG, HOB and MN, respectively. $x=(x_{1},x_{2},...,x_{n}) \in X \subset R^{n}$ is the n-dimensional feature vector.

### Multi-objective optimizationn

In order to solve the above six objective functions. The differential evolution operator is used to improve the crossover process of AGE-MOEA algorithm^14^ to enhance the global search ability of the algorithm. Finally, the improved algorithm is used to solve this problem. AGM-MOEA is a multi-objective optimization algorithm based on evolutionary algorithm, which performs well in exploring the Pareto surface with complex geometry. The specific calculation process of the improved AGE-MOEA algorithm is as follows.
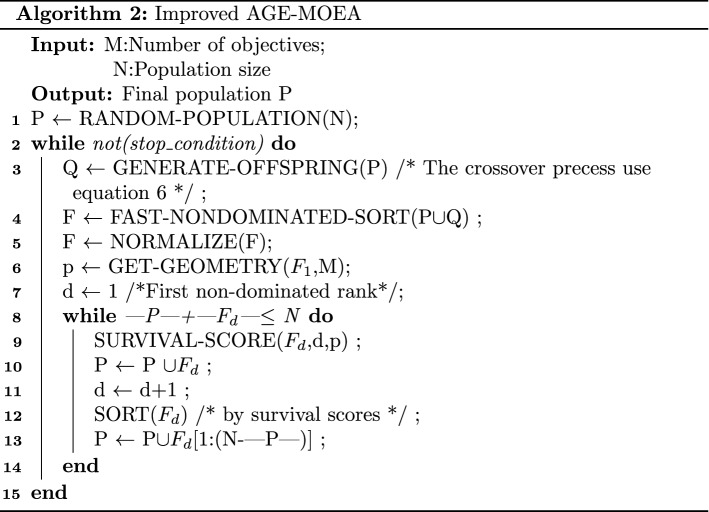


As shown in Algorithm 2, first, N populations are randomly initialized (line 2). Then, in each iteration process, the differential evolution operator is used for crossover, and the polynomial mutation operator is used for mutation to generate offspring (line 4). The resulting population is then divided into non-dominated levels(or fronts) using a non-dominated sorting algorithm^30^ (line 5) and normalized using the normalization method in NSGA-3^31^ (line 6). The value of the parameter p is calculated from the first non-dominated front ($F_{1}$) in each generation. Combined with the p value, the survival score is used to score the current non-dominated frontier from the aspects of diversity and proximity. By selecting the solution from the non-dominant frontier, a frontier (or level) is selected each time to form a new population of M solutions. The process terminates when the addition of solutions for the current non-dominant front end $F_d$ exceeds M. Finally, after sorting the survival scores in descending order, the remaining solutions are selected.

The differential evolution operator^35^ is used to replace the simulated binary crossover used in the original algorithm to generate the offspring(line 4 of the algorithm).

6 $$ {y}  = \left\{ {\begin{array}{*{20}l}    {x_{{r1}}  + F(x_{{r2}}  - x_{{r3}} ),} \hfill & {p < CR} \hfill  \\    {x_{{r1}} ,} \hfill & {otherwise} \hfill  \\   \end{array} } \right. $$

F and CR are two control parameters, where F = 1, CR = 0.7; the offspring is $y = ({y} _{1} ,...,{y} _{D} )$, D is the number of decision variables; $x_{r1},x_{r2},x_{r3}$ is three different parents; p is the random number of [0,1].

### Performance evaluation

To objectively measure the performance of the relational mapping model, we use mean squared error to evaluate the model’s performance on the $PIC_{50}$ regression prediction task, and accuracy to evaluate the model’s performance on the ADMET property regression prediction task.

Similarly, in order to objectively measure the performance of various multi-objective optimization algorithms, two indicators, inverted generational distance plus ($IGD^{+}$)^36^ and hypervolume (HV)^37^, are introduced here to evaluate the model search performance. The specific calculation method is as follows.

#### Inverted generational distance plus

Given a set of reference points $Z={z_{1},z_{2},...,z_{|M|}}$, where $z_{j}=(z_{j1},z_{j2},...,z_{jm})$ is the Pareto frontier point in the m-dimensional target space. Then $IGD^{+}(A,Z)$ is the average distance from each reference point $z_{j}$ to the nearest solution $a_{i} \in A$. In addition, the lower $IGD^{+}$ means that set A has better approximation along the Pareto front. The specific calculation formula is as follows.

7 $$\begin{aligned}{} & {} IGD^{+}(A,Z)=\frac{1}{|Z|}\sum _{j=1}^{|Z|}\min \limits _{a_{i} \in A}d_{IGD^{+}}(a_{i},z_{j}) \end{aligned}$$

8 $$\begin{aligned}{} & {} d_{IGD^{+}}(a_{i},z_{j}) = \sqrt{\sum _{k=1}^{m}(max(a_{ik}-z_{jk},0))^2} \end{aligned}$$

#### Hypervolume

For a m-objective space, HV represents the volume of a hypercube composed of each solution $a_{i}=(a_{i1},a_{i2},...,a_{im})$ and the reference point $z_{i}=(z_{i1},z_{i2},...,z_{im})$. In addition, the higher HV indicates that set A has better approximation along the Pareto front. Its specific definition is as follows.

9 $$\begin{aligned} HV(A,Z) = \lambda (\bigcup \limits _{a_{i} \in A}{[a_{i1},z_{1}]\times \cdot \cdot \cdot [a_{im},z_{m}]}) \end{aligned}$$

where $\lambda$ is the standard Lebesgue measure^38^. In this experiment, the reference point of HV is selected as the maximum value of Pareto frontier plus 1.

## Experimentation

### Experimental environment and model parameters

The hardware platform of this experiment is NVIDIA RTX3080 GPU, 12th Gen Intel(R) Core(TM) i7-12700KF CPU and 32GB RAM. The experimental software platform is Pychame 2019, and the algorithm libraries used are mainly Sklearn and Pymoo.

In this experiment, the number of clusters of spectral clustering used in feature selection is 25. A 10-fold cross-validation method was used to train and evaluate the relational mapping model. In solving the multi-objective optimization problem, the initial population size is 200, and the number of iterations is 210, which is consistent with Reference^28^. Crossover probability $p_c = 1$ in genetic operation ; mutation probability $p_m = 1/n$, n is the dimension of decision variable ; cross distribution index $\upnu _c = 20$; the variation distribution index $\upnu _m = 20$. Other parameters are consistent with those of the original algorithm.

### Introduction of data sets

The experimental data set was from the DrugBank molecular database of Alberta University^39^, including three files, namely ER$\alpha$_activity.xlsx, Molecular_ Descriptor.xlsx and ADMET.xlsx.

The ER$\alpha$_activity.xlsx file provided biological activity data for 1974 compounds against ER$\alpha$, including SMILES (Simplified Molecular Input Line Entry System). The biological activity value of the compound against ER$\alpha$ (expressed by $IC_{50}$, as the experimental measured value, the unit is nM, and the smaller the value is, the greater the biological activity is, and the more effective it is to inhibit the activity of ER$\alpha$) and the $PIC_{50}$ (i.e., the negative logarithm of $IC_{50}$) obtained by transforming the $IC_{50}$ value. This value is usually positively correlated with biological activity, that is, the greater the $PIC_{50}$ value indicates the higher the biological activity ; in the actual QSAR modeling, pIC50 is generally used to represent the biological activity value).

The Molecula_Descriptor.xlsx file gives 729 molecular descriptors (i.e. independent variables) for 1974 compounds. The first column is also the SMILES formula for compounds(numbered in the same order as above), followed by a total of 729 columns, each representing a molecular descriptor of the compound (i.e. an independent variable). Molecular descriptors of compounds are a series of parameters used to describe the structure and properties of compounds, including physical and chemical properties (such as molecular weight, LogP, etc.), topological structure characteristics (such as the number of hydrogen bond donors, the number of hydrogen bond acceptors, etc.), etc.

The ADMET.xlsx file provides data on the five ADMET properties of the above 1974 compounds. The first column is also the SMILES formula (the numbering order is the same as that before) representing the structure of the compound. The following five columns correspond to the ADMET properties of each compound, and the corresponding values are provided by the binary classification method. Caco-2: ’1’ represents the compound of intestinal epithelial cell permeability is good, ’0’ represents the compound of intestinal epithelial cell permeability is poor; cYP3A4: ’1’ means the compound can be metabolized by CYP3A4, ’0’ means the compound can not be metabolized by CYP3A4; hERG: ’1’ represents that the compound has cardiotoxicity, and ’0’ represents that the compound does not have cardiotoxicity; hOB: ’1’ indicates that the oral bioavailability of the compound is good, and ’0’ indicates that the oral bioavailability of the compound is poor; mN: ’1’ means the compound is genotoxic, ’0’ means the compound is not genotoxic.

### Experimental result

#### Feature selection

The feature selection algorithm proposed in this paper is used to run 10 times on the data set after data cleaning. The features with a frequency greater than 10 times are selected as the final candidate features, and 37 molecular descriptors are finally obtained. In order to more specifically reflect the specific meaning of the molecular descriptors selected, the classification of their pharmacological properties was counted here, and the results are shown in Table 1.

**Table 1:** Candidate molecular descriptor classification statistics.

Description types	Number	Description types	Number
Atom type electrotopological state	13	Chi cluster	3
Extended topochemical atom	4	Chi chain	3
Molecular linear free energy relation	2	ALOGP	2
Autocorrelation (mass)	1	Atom count	1
Carbon types	1	BCUT	1
Hbond acceptor count	1	Chi path cluster	1
Molecular distance edge	1	Largest Pi system	1
Ring count	1	XLogP	1

The electrotopological state index is a two-dimensional molecular descriptor based on the atomic level proposed by Kier and Hall^40^, the founder of molecular connectivity. It can simultaneously characterize the topological structure and electrical characteristics of compound molecules, and has been widely used in QSAR studies of drugs. Similar to the electrotopological state index, the extended topological chemical atom(ETA) index^41^ was proposed by Roy and Ghosh for the extension of the concept of topological chemical uniqueness of arrival(TAU) developed in the valence electron mobility (VEM) environment in the late 1980s. It is equally important in the study of toxicity and ecotoxicity modeling in the field of quantitative structure-activity relationships (QSARs). It can be seen from Table 1 that 13 molecular descriptors belong to the ’Atom type electrotopological state’ category, and 4 molecular descriptors belong to the ’Extended topochemical atom’ category, accounting for the first and second in the selected molecular descriptors, respectively. It can be seen that the features selected by the feature selection algorithm in this paper have certain rationality. In addition, the 37 molecular descriptors selected covered a total of 16 different categories of pharmacological properties. This shows that the pharmacological properties of the selected molecular descriptors cover a wide range. Compared with the original molecular descriptors, the selected molecular descriptors have rich information expression ability and better retain the important pharmacological properties of the original molecular descriptors.

In order to measure the pros and cons of more specific feature selection algorithm. In this paper, the selected molecular descriptors are used to combine the SVM algorithm for $PIC_{50}$ regression task and ADMET property classification task experiment, and the effect is compared with the feature selection method proposed by FSSC^21^ algorithm. The results are shown in Table 2.

**Table 2:** Comparison of feature selection algorithms.

Algorithm	$PIC_{50}$	AVG
FSSC	0.50	89.61
Ours	**0.48**	**89.73**

AVG:ADMET property prediction average accuracy.

Significant values are in [bold].

It can be seen from Table 2 that the performance of the selected features on the SVM algorithm is better than that of the FSSC algorithm after using the unsupervised feature selection algorithm proposed in this paper. The reason is that the FSSC algorithm uses features with higher variance and lower correlation as candidate features, which has limitations. When a feature in the original data is affected by noise, its variance often increases. With the increase of noise samples, it often interferes with the prediction performance of the model. At the same time, the lower correlation means that the relationship between the current feature and other features in the cluster is weaker. Using this feature to represent the candidate features of the entire cluster does not fully reflect the overall characteristics of the current cluster. Therefore, this paper first eliminates noise samples by data cleaning, and it is reasonable to use the most relevant features to represent the candidate features of the current cluster.

#### Relation mapping

The CatBoost algorithm^27^ is compared with a variety of traditional machine learning algorithms (SVM, AdaBoost, GBDT, XGBoost, RandomForest) for 10-fold cross validation and compared with the PsoBpSvm^26^ algorithm in the prediction of six optimization objectives. The results are shown in Table 3.

**Table 3:** Comparison of relation mapping models.

Algorithm	Index evaluation						
	$PIC_{50}$	Caco-2	$CYP_{3}A_{4}$	hERG	HOB	MN	AVG
SVM	0.48	89.43	91.47	90.07	85.21	92.46	89.73
AdaBoost	0.72	87.46	91.97	89.71	83.45	90.70	88.66
GBDT	0.53	90.42	92.88	90.77	85.84	93.09	90.60
XGBoost	0.53	89.36	92.39	89.50	83.94	91.90	89.42
RandomForest	0.50	90.35	93.02	90.91	86.69	94.50	91.09
PsoBpSvm	0.53	89.37	**94.06**	84.14	79.09	84.49	86.23
CatBoost	**0.44**	**91.12**	93.52	**91.33**	**86.83**	**94.85**	**91.53**

AVG:ADMET property prediction average accuracy.

Significant values are in [bold].

As can be seen from Table 3, using the features selected in the previous section for relational mapping experiments, the performance of the selected features on many machine learning algorithms is better than the PsoBpSvm algorithm. This again verifies the effectiveness of the feature selection method proposed in this paper. In addition, it can be seen that the CatBoost algorithm shows better prediction performance than other machine learning algorithms.

#### Target conflict analysis

**Figure 2:**
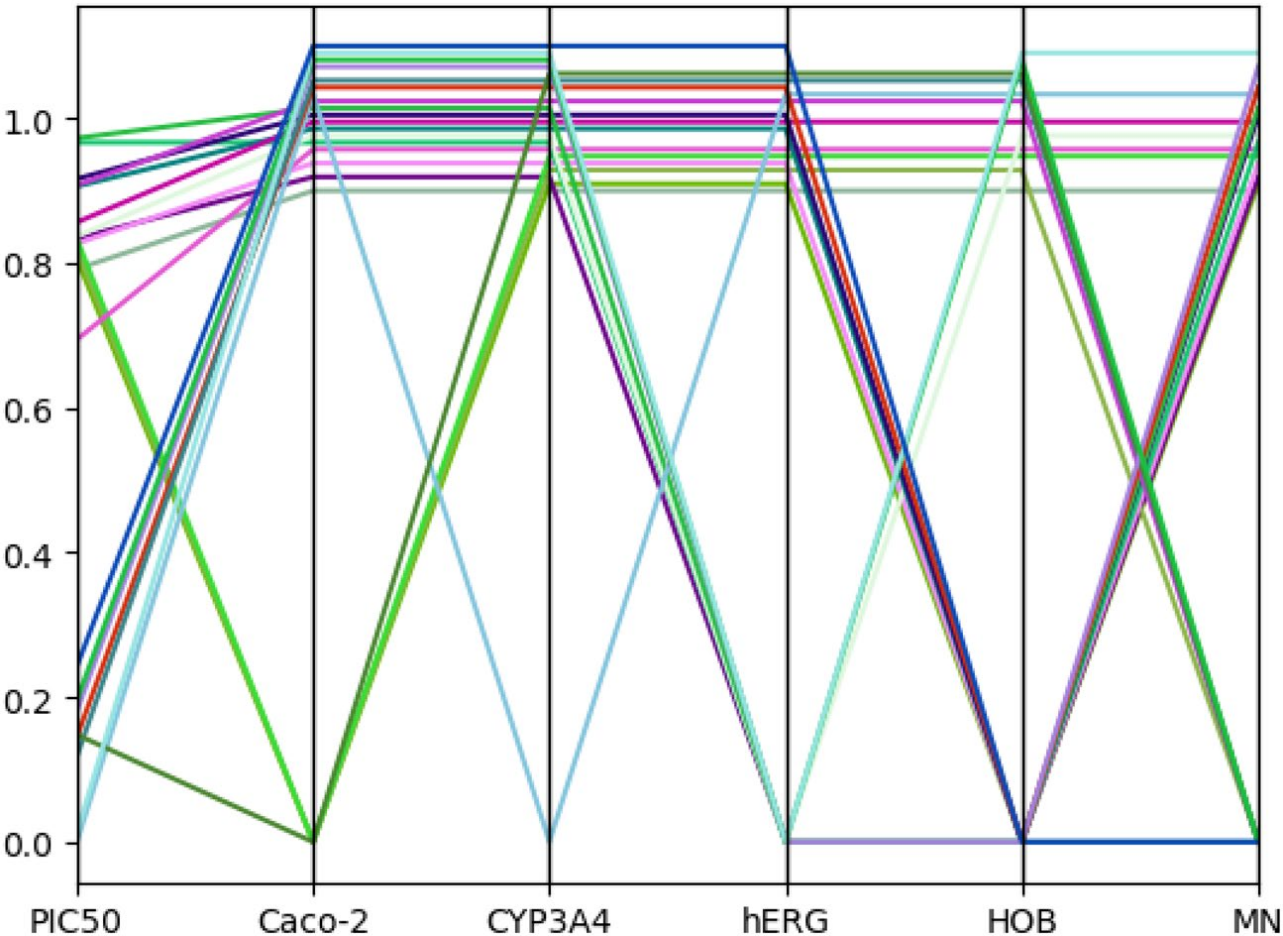
Optimization target conflict analysis.

Because in this problem, the Pareto front is unknown. Therefore, the treatment method of Reference^28^ is adopted here. A variety of optimization algorithms (NSGA-2^30^, NSGA-3^31^, AGE-MOEA^14^ and the algorithm in this paper) are used to run 10 times respectively. The calculated results are combined and de-duplicated as the approximate solution of the Pareto front of this problem. At the same time, in order to analyze the conflict relationship between the various objectives, this paper uses the parallel coordinate system^42^ used in^28^ to analyze the relationship between the various objectives. It should be noted that the parallel coordinate system is often used to visualize the values of each target to represent the correlation between the paired targets. The greater the slope of the line between one target and another, the greater the potential conflict between the two. The specific results are shown in Fig. 2. (Among them, normalization is used to scale the scale differences between different targets. At the same time, in order to prevent the overlap of lines from adversely affecting the observation, the data was scaled in the range of 0.9–1.1).

It can be seen from Fig. 2 that there is a large fluctuation in the slope of the connection between different targets. That is to say, among the six objectives to be optimized, there is both a competitive relationship and a mutual relationship between different objectives. Therefore, this problem is not easy to directly transform the multi-objective optimization problem into a single-objective optimization problem by weighting. In addition, weights are often harmful^43^.

**Figure 3:**
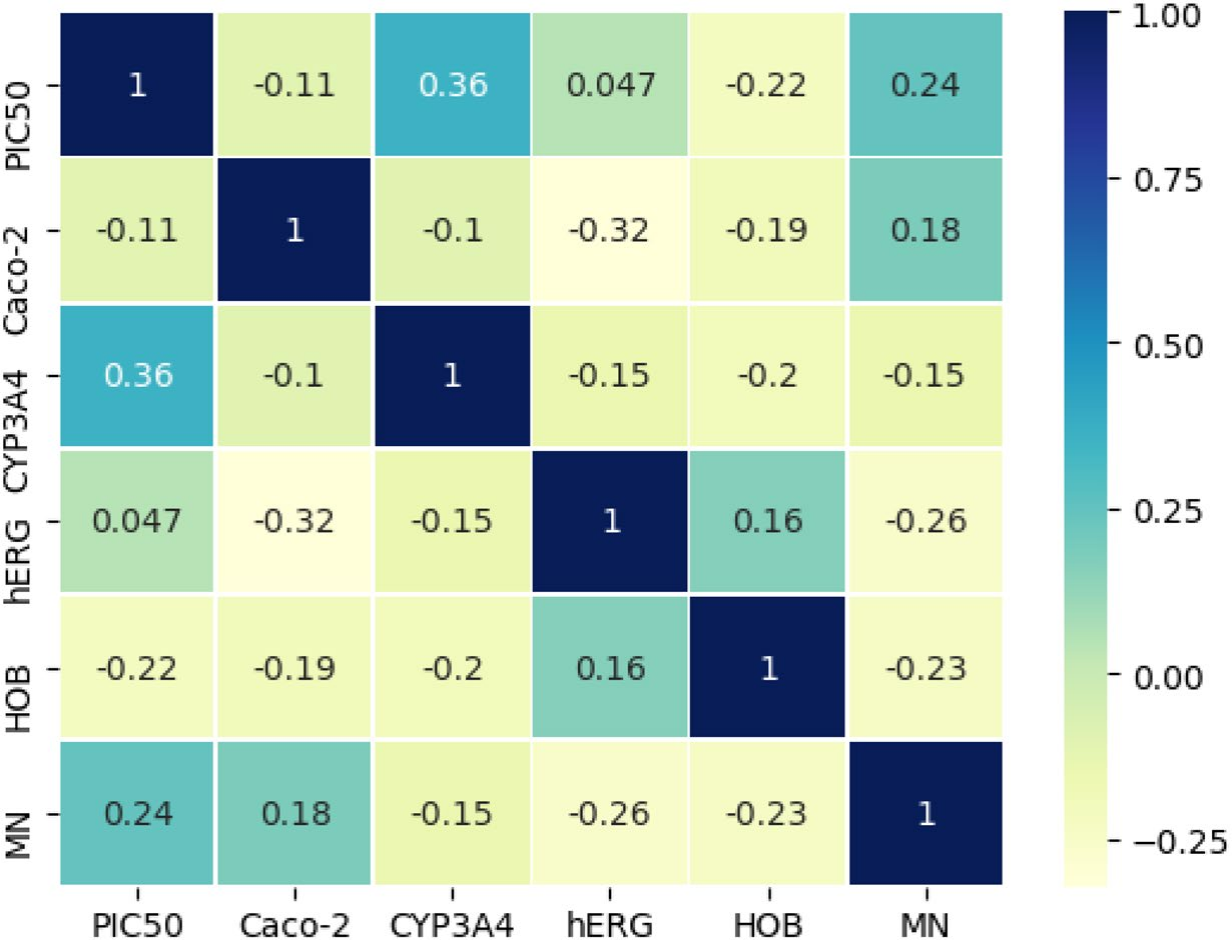
Optimization target correlation analysis.

To supplement the correlation analysis between objectives, we analyzed the correlation between objectives in the selected Pareto front approximation, as shown in Fig. 3.

It can be seen from Fig. 3 that there was a positive correlation between $PIC_{50}$ and $CYP_{3}A_{4}$, hERG, MN, and a negative correlation between $PIC_{50}$ and Caco-2, HOB. Therefore, in the optimization process of $PIC_{50}$, the optimization process of some targets will be suppressed, and the synchronous optimization between them cannot be realized. Similarly, there are similar conflict effects in the optimization process of other objectives.

#### Multi-objective optimization results

**Figure 4:**
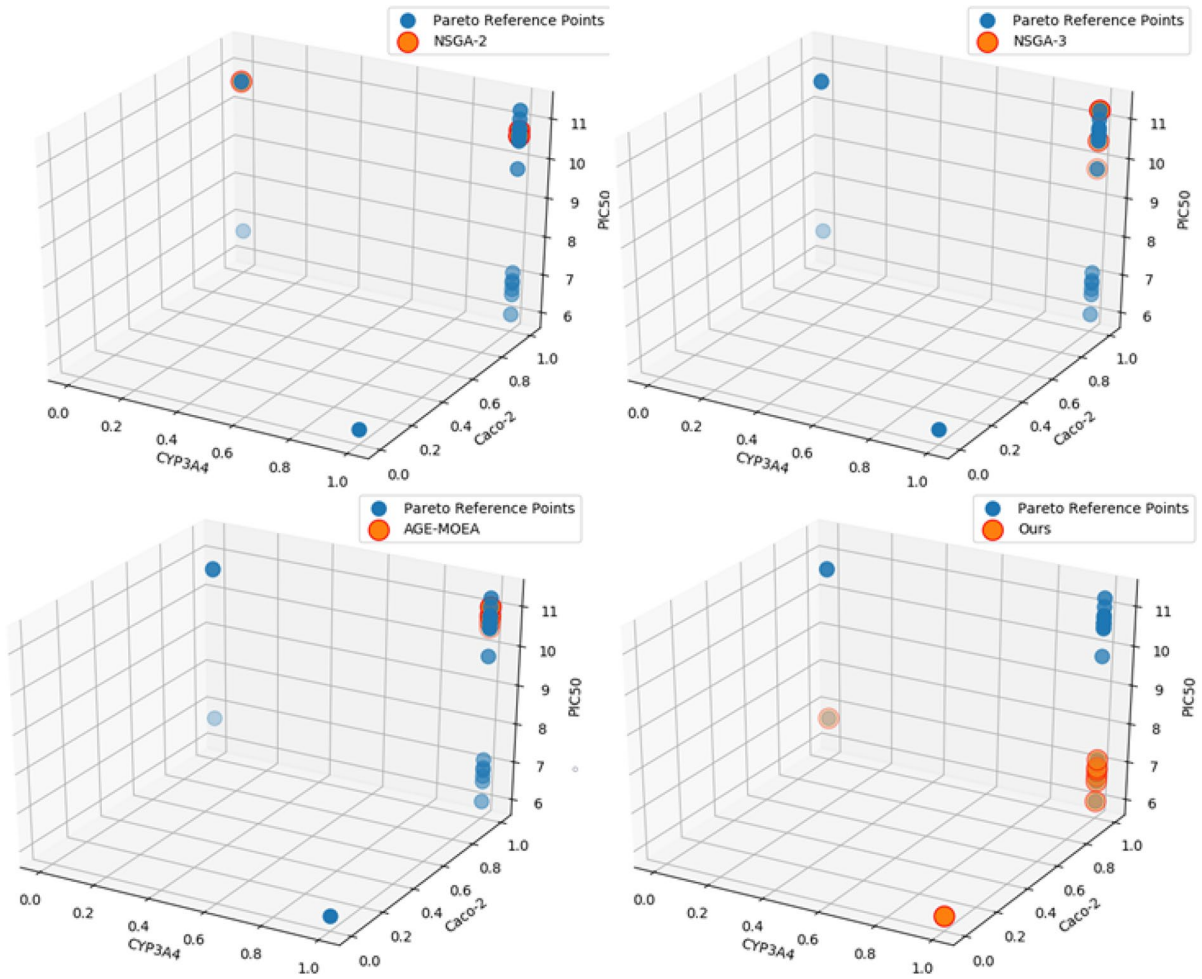
Visualization of solution results.

Due to the different correlation between the objectives, the complexity of the Pareto surface shape is determined. Through the previous analysis, it can be seen that there are both conflict and mutual promotion relationships among the six optimization objectives. It can be seen that the Pareto surface corresponding to this problem is more complex. Considering the advantage of AGE-MOEA algorithm in exploring complex Pareto surface shape, this paper chooses AGE-MOEA algorithm as the benchmark algorithm and improves it. At the same time, in order to visually display the results of various algorithms, $CYP_{3}A_{4}$, Caco-2 and $PIC_{50}$ are selected as X, Y and Z axes respectively. The solution results are compared with the approximate values of the Pareto front obtained above, and the specific results are shown in Fig. 4.

It can be seen from Fig. 4 that the algorithm in this paper has achieved more search results in solving this problem than the other three algorithms. At the same time, in order to measure the performance of various algorithms more specifically, objective evaluation indicators are selected for performance evaluation. The results are shown in Table 4.

**Table 4:** Comparison of optimization algorithms.

Algorithm	$IGD^{+}$	HV
NSGA-2	1.59	12.74
NSGA-3	1.59	6.21
AGE-MOEA	1.70	8.67
Ours	**0.04**	**57.17**

Significant values are in [bold].

It can be seen from Table 4 that the algorithm in this paper has achieved better performance than the other three multi-objective optimization algorithms, and its solution results have better approximation to the reference Pareto frontier.

After the above solution, the optimal range of 37 important molecular descriptors selected out in the anti-breast cancer candidate drugs can be obtained, which provides a direction for the selection of compounds. The specific value range is shown in Table 5.

**Table 5:** Molecular descriptor value range.

Molecular descriptors	Range	Molecular descriptors	Range
ALogP	[1.27, 4.89]	AMR	[123.17, 175.16]
nO	[3.25, 6.87]	ATSm1	[42.17, 53.10]
BCUTp-1h	[12.76, 15.41]	C3SP2	[3.45, 7.07]
SCH-5	[0.08, 0.31]	SCH-7	[0.66, 1.32]
VCH-6	[0.20, 0.43]	SC-3	[1.98, 2.96]
SC-4	[0.08, 0.27]	SC-5	[0.46, 1.00]
SPC-4	[5.21, 7.16]	nHBd	[1.67, 3.08]
nHBint5	[0.57, 1.97]	ndO	[0.87, 2.94]
SHBa	[37.23, 68.11]	SwHBa	[25.73, 39.62]
SHBint10	[4.42, 19.27]	minHssNH	[0.05, 0.46]
mindsCH	[0.23, 1.15]	minaaaC	[0.37, 1.32]
maxHBa	[12.12, 17.77]	maxwHBa	[2.04, 2.62]
maxHCsats	[0.46, 0.91]	maxaaCH	[2.04, 2.60]
ETA_Epsilon_1	[0.60, 0.68]	ETA_Beta_ns_d	[1.33, 2.22]
ETA_EtaP_F	[1.14, 1.31]	ETA_EtaP_B	[0.01, 0.02]
nHBAcc	[2.59, 6.91]	nAtomP	[16.66, 30.59]
MDEC-33	[10.26, 20.24]	MLFER_BH	[1.55, 2.74]
MLFER_S	[2.70, 3.68]	nRing	[3.99, 6.19]
XLogP	[2.99, 6.47]		

### Ablation experiment

#### Analysis of cluster number

In the selection of the number of clusters, it is usually based on the elbow rule or the contour coefficient. However, in this experiment, it was found that the effect of subsequent experiments using the features selected by such methods was poor. The reason is that the differences between features are small. Therefore, in this experiment, we will select the number of clusters according to the prediction performance of the SVM model after 10 cross-validations. The specific experimental results are shown in Table 6.

**Table 6:** Comparison of the number of clusters.

Clustering number	$PIC_{50}$	AVG
20	0.49	89.40
25	0.48	**89.73**
30	**0.47**	89.70

Significant values are in [bold].

It can be seen from Table 6 that when the number of clusters is 25, the feature mapping model has the best performance on the ADMET property. When the number of clusters increases to 30, the prediction performance of the model on ADMET properties decreases slightly, but the prediction performance on ADMET properties increases slightly. Considering that the increase of the number of features will increase the training cost of the model, but the performance difference is not large, the number of clusters selected in this experiment is 25.

#### Analysis of population initialization size

In order to analyze the influence of the initial population size on the algorithm, the initial population size is discussed here. The specific results are shown in Table 7.

**Table 7:** Comparison of initial population number.

Population size	$IGD^{+}$	HV
100	0.21	45.85
200	**0.04**	**57.17**
300	**0.04**	**57.17**

Significant values are in [bold].

It can be seen from Table 7 that when the population initialization scale is 200, the algorithm has reached convergence. Increasing the initial population size to 300 will not improve the performance of the algorithm. Therefore, in this experiment, the initial population size is 200.

## Conclusion

Aiming at the selection of anti-breast cancer candidate drugs, this paper proposes a complete drug selection framework from three aspects: feature selection, relationship mapping and multi-objective optimization. In the feature selection part, the relationship between features is explored from multiple perspectives of correlation coefficient, cosine similarity and gray correlation coefficient. Spectral clustering is used for feature clustering and a new feature importance measurement method is proposed to select the final intra-cluster features. In the relationship mapping part, a variety of machine learning algorithms are used for comparative experiments. Finally, the CatBoost algorithm is selected for relationship modeling, which achieves better prediction performance. In the multi-objective optimization part, based on the analysis of the conflict relationship between the objectives, the AGE-MOEA algorithm is improved. The improved algorithm is used to solve this problem, which achieves better search performance than many algorithms. We hope that the anti-breast cancer drug selection framework proposed in this paper can provide guidance for actual drug selection.

## Supplementary Information

Supplementary Information 1.

Supplementary Information 2.

Supplementary Information 3.

Supplementary Information 4.

## Data Availability

The data generated during this study are included in the article and its Supplementary information.
